# Health state utilities associated with post-surgical *Staphylococcus aureus* infections

**DOI:** 10.1007/s10198-019-01036-3

**Published:** 2019-03-18

**Authors:** Louis S. Matza, Katherine J. Kim, Holly Yu, Katherine A. Belden, Antonia F. Chen, Mark Kurd, Bruce Y. Lee, Jason Webb

**Affiliations:** 1 Evidera, 7101 Wisconsin Avenue, Suite 1400, Bethesda, MD 20814 USA; 2 Pfizer Inc, Collegeville, PA USA; 3 Sydney Kimmel Medical College, Thomas Jefferson University, Philadelphia, PA USA; 4 Department of Orthopaedics, Brigham and Women’s Hospital, Harvard Medical School, Boston, MA USA; 5 Department of Orthopedic Surgery Sidney Kimmel Medical College, Thomas Jefferson University The Rothman Institute, Philadelphia, PA USA; 6 Johns Hopkins University Bloomberg School of Public Health, Baltimore, MD USA; 7 Avon Orthopaedic Centre, Southmead Hospital, Bristol, UK

**Keywords:** Utility, Time trade-off, Surgical site infection, SSI, Surgery, Staphylococcus aureus, I00 [Health, Education, and Welfare (General)]

## Abstract

**Introduction:**

Surgical site infections (SSIs) are among the most common and potentially serious complications after surgery. *Staphylococcus aureus* is a virulent pathogen frequently identified as a cause of SSI. As vaccines and other infection control measures are developed to reduce SSI risk, cost-utility analyses (CUA) of these interventions are needed to inform resource allocation decisions. A recent systematic review found that available SSI utilities are of “questionable quality.” Therefore, the purpose of this study was to estimate the disutility (i.e., utility decrease) associated with SSIs.

**Methods:**

In time trade-off interviews, general population participants in the UK (London, Edinburgh) valued health states drafted based on literature and clinician interviews. Health states described either joint or spine surgery, with or without an SSI. The utility difference between otherwise identical health states with and without the SSI represented the disutility associated with the SSI.

**Results:**

A total of 201 participants completed interviews (50.2% female; mean age = 46.2 years). Mean (SD) utilities of health states describing joint and spine surgery without infections were 0.79 (0.23) and 0.78 (0.23). Disutilities of SSIs ranged from − 0.03 to − 0.32, depending on severity of the infection and subsequent medical interventions. All differences between corresponding health with and without SSIs were statistically significant (all *p* < 0.001).

**Conclusion:**

The preference-based SSI disutilities derived in this study may be used to represent mild and serious SSIs in CUAs assessing and comparing the value of vaccinations that may reduce the risk of SSIs.

**Electronic supplementary material:**

The online version of this article (10.1007/s10198-019-01036-3) contains supplementary material, which is available to authorized users.

## Introduction

Surgical site infections (SSIs) are among the most common and potentially serious complications after surgery [1]. *Staphylococcus aureus* (*S. aureus*), a human commensal and bacterial pathogen, is frequently identified as the cause of SSIs and can result in superficial skin infections as well as deeper tissue infections [2]. Post-surgical *S. aureus* infections are associated with potentially severe outcomes such as sepsis as well as longer hospital stays, dramatically increased economic burden, and a fivefold increased risk of in-hospital death [3–6].

As vaccines and other infection control measures are introduced to reduce SSI risk [3, 7, 8], economic modeling is needed to assess their value and inform resource allocation decisions [9]. A cost-utility analysis (CUA), a type of model that incorporates preferences for various treatment-related outcomes [10, 11], requires health state utilities to calculate quality-adjusted life years (QALYs). Utilities are values anchored to 0 (dead) and 1 (full health) that quantify the strength of preference for health states [12, 13].

Although previous studies have reported utilities for SSIs, the available utility values for SSIs have some notable limitations [14]. For example, some utilities are based on author assumptions rather than preference-based valuations [15–19]. One vignette-based valuation study estimated utilities for two health states describing infections following hip arthroplasty [20]. However, the infection health states provided very limited description of the infections and treatments, and they were presented as an ongoing chronic condition lasting 15 years. This timeframe is not consistent with the typical course of SSIs, which usually resolve in less than a year. Therefore, the resulting utilities are based on health states that may not be a clear or accurate representation of SSIs. Another study reported utilities for patients who completed a utility assessment years after the SSI [21]. The resulting utilities represent health states after the infection has resolved, rather than the SSI. Because of limitations in published SSI utilities, CUAs of SSI interventions [9, 22, 23] have often used utilities that were not originally derived to represent SSIs [24–26]. Furthermore, authors of a recent systematic review concluded that available SSI utilities have “questionable quality” [14]. In light of these findings, further research on SSI utility estimates is needed.

The purpose of this study was to estimate disutility (i.e., decrease in utility) associated with several types of post-surgical *S. aureus* infection. An SSI involves a series of temporary experiences including symptoms, antibiotic treatment, possible surgical intervention, additional hospital time, and extended recovery. Generic preference-based measures such as the EQ-5D estimate utility at one point in time, and they are not well-suited for capturing the utility impact of temporary health experiences that change over time. Therefore, the current study used a vignette-based approach to estimate SSI utilities.

## Methods

### Overview of study design

While SSIs can occur after any type of surgery, this study was designed to estimate the disutility of SSIs following either joint surgery (i.e., hip or knee) or spine surgery. These types of surgeries were selected to provide context for the SSI because SSIs following joint and spine surgeries are known to be associated with increased morbidity, mortality rates, healthcare resource utilization, and costs [5, 27].

Health states (i.e., vignettes) were developed based on published literature and clinician interviews and refined based on a pilot study. The health states described a 1-year period beginning with either joint or spine surgery, with or without a subsequent SSI. The utility difference between otherwise identical health states with and without an SSI represents the disutility of the SSI. Health states that change over time are called path states, and utilities are estimated for the whole path rather than each part of the path [28–30].

Utilities for these health states were elicited in time trade-off (TTO) interviews with general population participants in London and Edinburgh, UK. Participants provided written informed consent, and procedures were approved by an independent Institutional Review Board (Ethical and Independent Review Services; Study Number 16023).

### Health state development

Literature review was conducted to ensure the health states were consistent with published research and inform the development of a clinician interview guide. This literature review focused on hip and knee replacement and spine surgery [31–36]; SSIs [31, 37–42]; interventions for SSIs [37, 38, 40, 41, 43–45]; and duration of SSI treatment and recovery [39, 41, 44, 46].

Multiple rounds of telephone interviews were conducted with seven clinicians: two spine surgeons, two surgeons specializing in hip and knee arthroplasty, two infectious disease specialists, and one general practice physician with experience in utility assessment associated with SSIs. The four US clinicians had MD degrees. Degrees of the three UK clinicians were MBChB/FRCS/MD, BM BCh, and BSc/MBBS. Each clinician participated in multiple discussions.

Health states were developed through an iterative process with the clinicians, and each clinician participated in up to seven discussions so that they could respond to drafts of the health states as they developed. On one occasion, five of the clinicians joined a teleconference to come to a consensus on how to best represent and describe the most typical interventions for SSIs. When there was a discrepancy between US and UK language or treatment patterns, it was determined that the health states would represent the UK approach because the valuation study was planned for a UK general population sample.

Seven health states were drafted, each describing 1 year of a patient’s life beginning with surgery. Health states A and E described a year beginning with joint (A) or spine (E) surgery without an SSI, followed by gradual recovery and return to normal functioning. The other health states started with the same descriptions as A and E, followed by added descriptions of SSIs based on clinicians’ reports of the typical course and treatment. The specific types of SSI and associated treatments were selected based on clinicians’ reports of the most common course and treatment of SSIs associated with joint and spine surgery.

Three health states (B, C, D) began with joint surgery (same as A), followed by an SSI. The joint was unspecified so that disutilities could apply to SSIs following either hip or knee surgery. The clinicians explained that SSIs resulting from these two surgeries led to similar symptoms and interventions, and therefore did not require separate health states. Health state B described a superficial wound infection treated with antibiotics. Health state C described a deep infection treated with antibiotics and a second surgery involving debridement and implant retention (DAIR). Health state D described a deep infection treated with antibiotics and a series of two additional surgeries (the first to remove infected tissue and insert a temporary joint implant; the second to remove additional infected tissue and insert a permanent implant).

Two health states (F, G) began with spine surgery (same as E), followed by an SSI. Health state F described an infection treated with antibiotics. Health state G described a deeper infection requiring antibiotics and an additional surgery to remove infected soft tissue. Because surgery for SSI following spine surgery never involves removal of surgical hardware (unlike joint surgery), clinicians advised that one health state would be sufficient to capture SSI requiring surgery.

Health states were presented on individual cards, each with a series of bullet point descriptions categorized with headings to help respondents understand the sequence of events. For example, headings for health state A were: surgery, hospital stay, after surgery, and recovery. Health state B added two headings: infection and treatment of infection. To help respondents understand the sequence of events, a timeline was depicted at the bottom of each health state card. See the electronic supplementary material for health state text.

### Participants

General population participants were required to be at least 18 years old; able to understand interview procedures; and a UK resident. No clinical characteristics were required because this study aimed to estimate utilities for CUAs in submissions to health technology assessment agencies, which often prefer utilities representing general population values [47–49]. Participants were recruited via newspapers and online advertisements.

### Pilot study

To assess the clarity of health states and finalize the choice of utility assessment methodology, a pilot study was conducted in London with 18 general population participants (55.6% male; mean age = 36.2 years; age range = 21–56 years). Participants consistently reported that the health states were clear and comprehensible, although minor formatting and text edits were made based on specific comments. In addition, the TTO time horizon was varied so that the optimal time horizon could be selected for the subsequent valuation study (as discussed in the section titled “TTO Time Horizon” below).

### Utility interview procedures and scoring

As an introductory task prior to the utility valuation, participants were asked to rank the seven health states. To control for order effects, participants were randomized to review either the group of joint surgery health states or spine surgery health states first, followed by the other group. Within the joint surgery group and the spine surgery group, the health states were presented in random order. Along with the health states, participants were shown a background information page briefly describing the medical condition requiring surgery. Background for joint surgery was: “At one of the joints in your lower body, the cartilage covering the bone had worn down. This caused bones to rub together, which was painful.” Background for spine surgery was “You have arthritis in your spine which caused the nerves to be pinched. This pressure on the nerves caused persistent pain in your legs. You also had back pain from the arthritis.”

After the ranking, participants valued the health states in a TTO task with a 1-year time horizon and 1-month trading increments. Participants were offered a choice between living 1 year in the health state being rated or a shorter duration in full health. Choices alternated between longer and shorter amounts of time in full health, specified in months: 12, 0, 11, 1, 10, 2, 9, 3, 8, 4, 7, 5, and 6. For health states perceived as better than dead, utility scores (*u*) were calculated based on the point of indecision as the number of months in full health (*x*) divided by the number of months in the health state being rated (*u* = *x*/12 months), yielding a utility on a scale with the anchors of dead (0) and full health (1).

When participants perceived a health state as worse than dead, the task and scoring procedures were altered as described in previous literature [50, 51]. Participants were offered a choice between dead (choice 1) and a 1-year life span (choice 2) beginning with varying amounts of time in the health state being rated, followed by full health for the remainder of the 1-year life span. The resulting negative utility scores were calculated as *u* = −*x*/12, where *x* is the number of months in full health, and 12 is the number of months in the total life span of choice 2.

### TTO time horizon

In TTO valuations, the duration of time in the health state being rated (i.e., the time horizon) varies across studies, and this time horizon may have an impact on results [52, 53]. In the pilot study, each participant valued the health states in a TTO task with three time horizons: (1) 1 year with 1-month trading increments; (2) 2 years with 2-month trading increments; and (3) 10 years with 6-month trading increments. The order of the time horizons was varied to avoid order effects. The 10-year time horizon appeared to have a ceiling effect. Because the SSIs only occurred during the first year of the 10-year time period, many participants were not willing to trade time from this longer lifespan to avoid the temporary SSIs. Thus, the 10-year approach was not sensitive to differences in preference related to SSIs.

The 1-year and 2-year time horizons yielded almost identical patterns of utility scores, but the 1-year time horizon was preferable for two reasons. First, the 1-year time horizon yields disutilities that can be used in models as a QALY decrement without further calculations. Second, SSIs generally resolve in less than 3–6 months. Therefore, the shorter time horizon is sufficient for capturing the SSIs without excessive time following the events, allowing respondents to focus on the SSI. Based on these pilot study results, the subsequent valuation study was conducted with the 1-year time horizon.

### Statistical analysis procedures

Analyses (SAS version 9.4) were primarily descriptive (e.g., means and standard deviations). SSI disutilities were calculated by subtracting the utility of health states without SSIs (A and E) from the utility of corresponding health states with SSIs (B, C, D, F, G). Demographic subgroups (age, gender, geographic location) were compared with Chi square analyses (for categorical variables) and t-tests (for continuous variables). Pairwise t-tests were conducted to test whether there were significant differences between health states with and without SSIs.

## Results

Of the 213 participants who attended interviews, 12 were unable to complete the utility assessment procedures due to insufficient comprehension of the health states or assessment procedures during the introductory ranking or TTO procedure (some of these 12 respondents asked to discontinue the interview, while others provided only illogical responses even after the interviewer made multiple attempts to clarify the task). Therefore, the analysis sample included 201 participants (98 Edinburgh; 103 London; 50.2% female; mean age = 46.2 years). Participant characteristics are summarized in Table 1. The only statistically significant difference between the Edinburgh and London subgroups was that a greater percentage of participants in Edinburgh reported ethnic/racial background as white (92.9% vs. 64.1%; *p* < 0.0001).

**Table 1:** Participant characteristics

Characteristics	Edinburgh (*N* = 98)	London (*N* = 103)	Total sample (*N* = 201)	*p* value^a^
Age (mean, SD)	45.2 (17.6)	47.1 (14.2)	46.2 (15.9)	0.42
Gender (*n*, %)				
Male	50 (51.0%)	50 (48.5%)	100 (49.8%)	0.73
Female	48 (49.0%)	53 (51.5%)	101 (50.2%)	
Ethnic/racial background (*n*, %)				
White	91 (92.9%)	66 (64.1%)	157 (78.1%)	< 0.0001
Mixed	3 (3.1%)	5 (4.9%)	8 (4.0%)	
Asian	4 (4.1%)	15 (14.6%)	19 (9.5%)	
Black	0 (0.0%)	15 (14.6%)	15 (7.5%)	
Other^b^	0 (0.0%)	2 (1.9%)	2 (1.0%)	
Marital status (*n*, %)				
Not married^c^	49 (50.0%)	57 (55.3%)	106 (52.7%)	0.45
Married/cohabitating/living with partner	49 (50.0%)	46 (44.7%)	95 (47.3%)	
Employment status (*n*, %)				
Full-time work	38 (38.8%)	41 (39.8%)	79 (39.3%)	0.13
Part-time work	21 (21.4%)	33 (32.0%)	54 (26.9%)	
Other^d^	39 (39.8%)	29 (28.2%)	68 (33.8%)	
Education level (*n*, %)				
University degree	40 (40.8%)	44 (42.7%)	84 (41.8%)	0.78
No university degree	58 (59.2%)	59 (57.3%)	117 (58.2%)	
Experience with any surgery (*n*, %)				
Yes	55 (56.1%)	54 (52.9%)	109 (54.5%)	0.65
No	43 (43.9%)	48 (47.1%)	91 (45.5%)	
If yes, type of surgery (*n*, %)				
Hip surgery	0 (0.0%)	1 (1.9%)	1 (0.9%)	0.25
Knee surgery	7 (12.7%)	2 (3.7%)	9 (8.3%)	
Spine surgery	3 (5.5%)	2 (3.7%)	5 (4.6%)	
Other	45 (81.8%)	49 (90.7%)	94 (86.2%)	

*SD* standard deviation

^a^*p* values are based on *t*-tests for continuous variables and Chi square analyses for categorical variables

^b^Other ethnic/racial background as reported by respondents includes “Arab” (1) and “Argentinian” (1)

^c^Not married includes single, divorced, separated, and widowed as reported by the respondents

^d^Other employments include homemaker, student, unemployed, retired, disabled, caregiver, and unspecified as reported by the respondents

The most commonly reported health conditions were anxiety (16.9%), depression (15.4%), arthritis (9.0%), diabetes (9.0%), and hypertension (8.0%). To ascertain relevant experience with the health state content, participants were asked whether they had experienced surgery. A total of 54.5% (*n* = 109) of the sample reported having surgery (56.1% Edinburgh; 52.9% London). Of the 109 respondents who reported having some type of surgery, 5 (4.6%) reported spine surgery, 9 (8.3%) reported knee surgery, and 1 (0.9%) reported hip surgery, while the other 94 (86.2%) had experienced surgery that was not described in the health states.

### Health state utilities

In the introductory ranking task, health states with more serious SSIs were consistently ranked as less preferable. These rankings ranged from 1 (most preferable health state) to 7 (least preferable health state). The joint surgery health state without an SSI (health state A) had a mean ranking of 1.5, followed by E (1.7), B (3.4), F (3.5), G (5.4), C (5.6), and D (7.0).

**Fig. 1:**
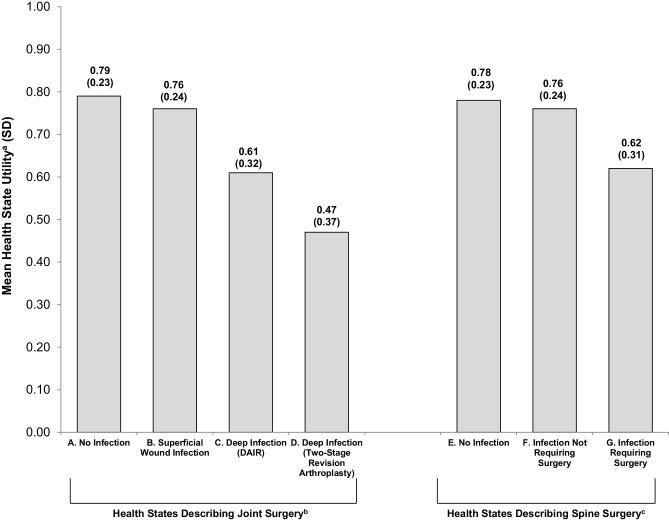
Health State Utilities. ^a^Utility scores are on a scale anchored with 0 representing dead and 1 representing full health. ^b^Health states B, C, and D include health state A, plus a description of infection. ^c^Health states F and G include health state E, plus a description of infection. *DAIR* debridement and implant retention

The two health states describing joint (A) and spine surgery (E) without an SSI had similar mean utilities of 0.79 and 0.78, respectively (Fig. 1). The addition of an SSI resulted in lower utilities for health states B, C, D, F, and G. T-tests comparing health state utilities found that all health states with SSIs had significantly lower utilities than the corresponding health state without an SSI (all *p* < 0.0001). For each individual respondent, the utility of health state A was greater than or equal to the utilities of health states B, C, and D. Similarly, health state E always received a utility score that was greater than or equal to those for health states F and G.

Disutilities (Table 2) were relatively small for SSIs that could be treated with antibiotics rather than surgery: − 0.030 for health state B and − 0.026 for health state F (three decimal places, rather than two, are provided here to show that these disutilities were not identical). However, the magnitude of utility increased substantially with deeper infection requiring additional surgery: − 0.16 for SSI plus surgical intervention following spine surgery, − 0.18 for SSI plus DAIR following joint surgery, and − 0.33 for SSI plus two-stage revision arthroplasty following joint surgery.

**Table 2:** Disutilities of surgical site infections (*N* = 201)

Health state differences	Mean	SD	95% CI
Joint surgery health states^a^			
B—A: superficial wound infection	− 0.03	0.08	− 0.04 to − 0.02
C—A: deep infection followed by DAIR	− 0.18	0.20	− 0.21 to − 0.15
D—A: deep infection (two-stage revision arthroplasty)	− 0.32	0.28	0.36 to − 0.29
Spine surgery health states^b^			
F—E: infection not requiring surgery	− 0.03	0.07	− 0.04 to − 0.02
G—E: infection requiring surgery	− 0.16	0.18	− 0.19 to − 0.14

*CI* confidence interval, *DAIR* debridement and implant retention, *SD* standard deviation

^a^Disutilities for surgical site infections (SSIs) associated with joint surgery were computed by subtracting the utility of health state A from the utility of health states B, C, and D. Health states B, C, and D were all identical to health state A, except for the addition of the SSI and associated treatments

^b^Disutilities for SSIs associated with spine surgery were computed by subtracting the utility of health state E from the utility of health states F and G. Health states F and G were identical to health state E, except for the addition of the SSI and associated treatments

Most respondents rated every health state as better than dead (i.e., utility score > 0). Health states A and E were rated as worse than dead by only 1 (0.5%) of the 201 respondents, and health states B and F were rated as worse than dead by only 2 (1.0%) respondents. The health states describing SSI with surgical intervention were rated as worse than dead slightly more often: G (*n* = 5; 2.5%), C (*n* = 6; 3.0%), and D (*n* = 12; 6.0%). T-tests found no statistically significant differences in utility or disutility between men and women; between older and younger respondents (categorized by median split); or between respondents from London and Edinburgh.

## Discussion

Results of the current study address some limitations of previously published utilities used to represent SSIs in economic modeling [14]. In this utility valuation study, SSIs were associated with statistically significant decreases in utility. Disutility was greater for health states describing more severe infections that require more invasive interventions. For example, the addition of a superficial wound infection following joint surgery resulted in a relatively small disutility of only − 0.03, while SSIs requiring one or two additional surgeries had disutilities of − 0.18 and − 0.33, respectively.

The disutilities derived in this study may be used to adjust QALYs in CUAs comparing interventions intended to reduce the risk of post-surgical infections. When using these utility scores, modelers need to consider the time horizon of the health states and TTO task. Each health state described 1 year that included a sequence of health-related events, and the TTO valuation was conducted with a 1-year time horizon. Therefore, the disutilities should be used to adjust a 1-year period of a model in which the SSI occurs. This could be done by applying QALY decrements. For example, a QALY decrement of − 0.03 (i.e., the disutility of health state B) would be applied to represent the utility impact of an SSI requiring oral antibiotics following joint surgery.

Health states that change over time have been called path states. Path states describe the experience of a hypothetical patient who proceeds through a sequence of different health states [28–30]. For example, health states B, C, D, F, and G begin with surgery, followed by an SSI, treatment for the SSI, and gradual recovery. The path state approach is useful for valuing health conditions that change over time because the states can be designed to represent the typical course of a medical condition and its treatment. A path state allows respondents to consider the sequence as a whole, as well as the duration of time spent in each part of the path. However, a limitation is that it is not possible to determine the utility impact of each event within the path. The disutilities derived in this study represent the overall utility decrease during a 1-year period in which the SSI occurs. These disutilities cannot be used to represent a part of this sequence.

Because SSIs change over time, standardized generic preference-based measures such as the EQ-5D would not be appropriate for estimating their disutility. These generic measures are designed to quantify health status at one point in time, which means they cannot capture the utility impact of the full SSI experience, including subsequent treatment and gradual recovery. In contrast, the vignette approach is well-suited for this purpose because health state vignettes can describe a sequence of health-related events. However, the results should be interpreted with caution due to the inherent limitations of vignette-based methods. For example, the resulting utility scores represent the specific health states, which are based on literature review and clinicians’ descriptions of a typical patient rather than an actual patient sample. Therefore, the extent to which these utilities are comparable to values that may be reported by actual patients is not known. Given the challenges of assessing utilities with patients at multiple time points during the SSI treatment and recovery process, it may not be feasible to collect utilities from actual patients to represent the full experience of an SSI. Still, when modelers use the current vignette-based disutilities to adjust utilities gathered from patient samples, they should be aware that utilities estimated with different methods may not be entirely comparable to each other.

Another limitation is that, while SSIs can occur in any location of the body, the health states described SSIs only in the context of joint and spine surgery. The interventions for SSI vary according to the location of the surgery. For example, clinicians interviewed for this study reported that SSIs following joint and spine surgery differed from each other with regard to both treatment and recovery period. Furthermore, authors of a recent systematic review suggested that utility estimates were needed for SSIs following non-orthopedic as well as orthopedic surgery. While the experience of an SSI following joint or spine surgery may be similar to SSI following types of non-orthopedic surgery, there could be differences for some patients. Therefore, the current utilities do not necessarily generalize to SSIs that may occur after surgeries in locations other than joints or the spine. Before applying the current disutilities to represent SSIs following other types of surgery, it is recommended that modelers consult with clinicians to examine the extent to which the current health states can be considered a reasonable approximation of SSIs occurring in other contexts.

There may also be limitations associated with characteristics of the sample. Data were collected in only two cities in the UK, and therefore, results cannot be considered truly nationally representative. However, efforts were made during sample recruitment to ensure that no particular demographic group (age, gender, racial/ethnic background, employment status) was over-represented relative to the UK general population.

Despite limitations, results of the current study will be useful in economic modeling of interventions for SSIs. This study provides preference-based disutility values to represent both mild and serious SSIs as complications of either joint or spine surgery. These values may be used to adjust QALYs in CUAs conducted to inform resource allocation decisions regarding interventions, such as vaccinations, that may reduce the risk of SSIs.

## Electronic supplementary material

Below is the link to the electronic supplementary material.

Supplementary material 1 (DOCX 99 KB)
