# Supplementary material for: Health state utilities associated with post-surgical Staphylococcus aureus infections
Source: Eur J Health Econ. 2019 Mar 18;20(6):819–27. doi: 10.1007/s10198-019-01036-3 (PMC6652168; doi:10.1007/s10198-019-01036-3)

Supplementary Material

Appendix A. Health State Text

**(For peer review purposes and possible web publication)**

**Health State A. Joint: No Infection**

1. **Surgery**

- You have **joint surgery** to address this problem.
- During surgery, the **surface of the bone is replaced** with an implant (made of metal, plastic, and/or ceramic).

1. **Hospital Stay**

- You stay in the hospital for **2 to 4 days** after the operation.
- During your stay, you receive **physiotherapy and occupational therapy** to help you regain mobility and the ability to care for yourself after you leave the hospital.

1. **After Surgery**

- You have **pain** from the surgery that **gradually decreases** for about a month.

1. **Recovery**

- After surgery, you use **crutches or sticks** **for 6 weeks**, and you continue physiotherapy exercises.
- **Six weeks** after surgery, you can **walk** **independently**, and you have **less pain** in your joint.
- You continue to improve. **Six months** after surgery, you have **normal functioning** for your age, but you occasionally have **minor pain** in the joint where the surgery occurred.

1. **Timeline**


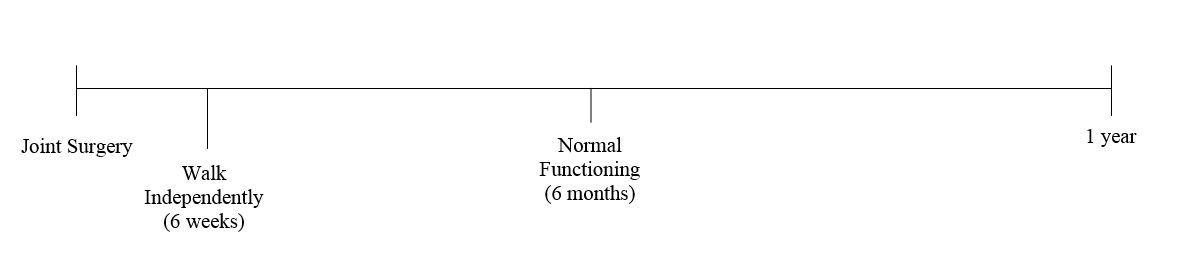


**Health State B. Joint: Superficial Wound Infection**

1. **Surgery**

- You have **joint surgery** to address this problem.
- During surgery, the **surface of the bone is replaced** with an implant (made of metal, plastic, and/or ceramic).

1. **Hospital Stay**

- You stay in the hospital for **2 to 4 days** after the operation.
- During your stay, you receive **physiotherapy and occupational therapy** to help you regain mobility and the ability to care for yourself after you leave the hospital.

1. **After Surgery**

- You have **pain** from the surgery that **gradually decreases** for about a month.

1. **Infection**

- Two weeks after surgery, you are diagnosed with a **bacterial infection** **of the skin**.
- You have **redness** **and swelling** on the skin surface where the surgery occurred. This area is **hot to the touch**.
- Your wound is **painful** **and sore** at the infection site.

1. **Treatment of Infection (Oral Antibiotics)**

- To **treat** this infection, you take **oral antibiotics** multiple times each day for two weeks.
- For some people, these antibiotics can have many **side effects** including diarrhoea, nausea, and allergic reactions.

1. **Recovery**

- After surgery, you use **crutches or sticks for 6 weeks**, and you continue physiotherapy exercises.
- **Six weeks** after surgery, you can **walk independently**, and you have **less pain** in your joint.
- You continue to improve. **Six months** after surgery, you have **normal functioning** for your age, but you occasionally have **minor pain** in the joint where the surgery occurred.

1. **Timeline**


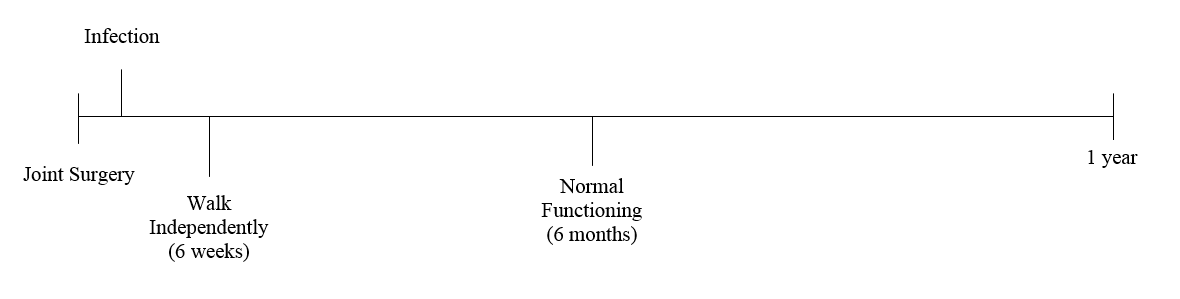


**Health State C. Joint: Deep Infection (Debridement and Implant Retention; DAIR)**

1. **Surgery**

- You have **joint surgery** to address this problem.
- During surgery, the **surface of the bone is replaced** with an implant (made of metal, plastic, and/or ceramic).

1. **Hospital Stay**

- You stay in the hospital for **2 to 4 days** after the operation.
- During your stay, you receive **physiotherapy and occupational therapy** to help you regain mobility and the ability to care for yourself after you leave the hospital.

1. **Infection**

- **Less than one month** after surgery, you are diagnosed with a **bacterial infection of the bone and soft tissue**.
- Because of this infection, you have significantly **increased pain** for about a month.
- You have **redness, swelling, and draining fluid** where the surgery occurred. This area **hot to the touch**.
- The **fluid draining** from the infection **stains** your clothing and bedsheets.

1. **Treatment of Infection (Second Surgery, IV Antiobiotics, and Oral Antibiotics)**

- You need **one additional surgery** to treat the infection.

**SECOND SURGERY**

- During surgery, **soft tissue** that appears **infected** is **removed**. **Parts of the implant** inserted during the initial surgery are **removed and replaced**.
- You stay in the hospital for **10 to 14 days**.

**MEDICATION AFTER SECOND SURGERY**

- In the hospital, you receive **antibiotic treatment** about 4 times per day by **intravenous (IV) infusion** in the hospital. This means it is administered into the bloodstream through a tiny plastic tube inserted directly into a vein. This takes about 30 minutes.
- After leaving the hospital, you continue to receive antibiotic treatment by IV once per day for 6 weeks. A **district nurse** will come to your home to administer treatments, which takes approximately 1 hour.
- You take **oral antibiotics** for 3 to 6 months.
- For some people, these antibiotics can have many **side effects** including diarrhoea, nausea, and allergic reactions.

1. **Recovery**

- After the first surgery, you use **crutches or sticks for about 3 months**, and you continue physiotherapy exercises.
- **Three months** after your original surgery, you can **walk independently**, and you have **less pain** in your joint.
- You continue to improve. **Eight months** after the original surgery, you have **normal functioning** for your age, but you occasionally have **minor pain** in the joint where the surgery occurred.

1. **Timeline**


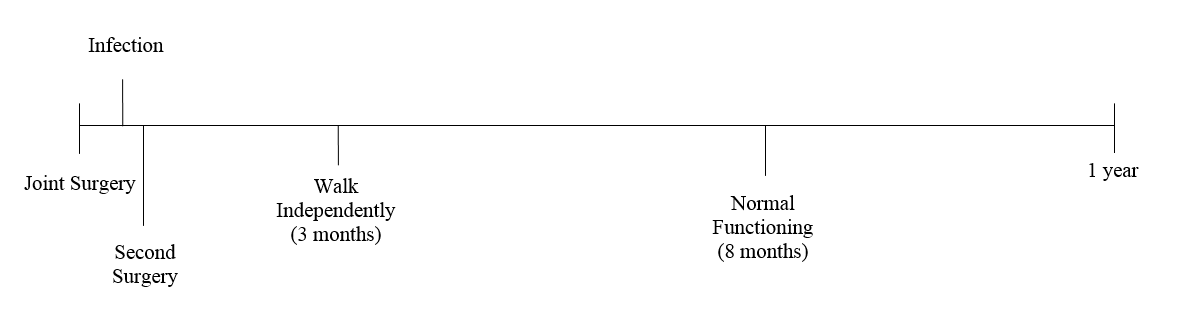


**Health State D. Joint: Deep Infection (Two-Stage Revision Arthroplasty)**

1. **Surgery**

- You have **joint surgery** to address this problem.
- During surgery, the **surface of the bone is replaced** with an implant (made of metal, plastic, and/or ceramic).

1. **Hospital Stay**

- You stay in the hospital for **2 to 4 days** after the operation.
- During your stay, you receive **physiotherapy and occupational therapy** to help you regain mobility and the ability to care for yourself after you leave the hospital.

1. **Infection**

- **Several months** after your surgery, you are diagnosed with a **persistent bacterial infection of the bone and soft tissue**.
- Because of this infection, you have **significantly** **increased, deep, and persistent pain** for over 2 months.
- You have **redness and swelling** around the joint where the surgery occurred. This area is **hot to the touch**.

1. **Treatment of Infection (Second Surgery, Third Surgery, and IV Antibiotics)**

- You need **two additional surgeries** to treat the infection.

**SECOND SURGERY**

- During this surgery, **soft tissue** that appears infected is **removed**. **The implant** that was inserted during the initial surgery is **removed and replaced** with a **temporary implant**. The temporary implant **delivers antibiotics** to the joint to treat the infection for 6 weeks.
- You stay in the hospital for **10 to 14 days**.

**MEDICATION AFTER SECOND SURGERY**

- In the hospital, you receive **antibiotic treatment** about 4 times per day by **intravenous (IV) infusion** in the hospital. This means it is administered into the bloodstream through a tiny plastic tube inserted directly into a vein. This takes about 30 minutes.
- After leaving the hospital, you continue to receive antibiotic treatment by IV once per day for 6 weeks. A **district nurse** will come to your home to administer treatments, which takes approximately 1 hour.
- Because there is a temporary implant, **walking is difficult** for you. Therefore, you require **pain medication**.

**THIRD SURGERY**

- About 8 weeks after the second surgery, you have a **third surgery**. During this surgery, any **remaining tissue** that appears infected **is removed**.
- Then, the temporary implant that was inserted during the second surgery is **removed and replaced** with a **permanent implant**.

**MEDICATION AFTER THIRD SURGERY**

- In the hospital, you receive **antibiotic treatment** by intravenous (IV) infusion for 2 days after surgery.
- You stay in the hospital for **7 to 10 days**.

1. **Recovery**

- After the first surgery, you use **crutches or sticks for about 6 months**, and you continue physiotherapy exercises.
- **Six months** after your original surgery, you can **walk independently**, and you have **less pain** in your joint
- You continue to improve. **Ten months** after the original surgery, you have **normal functioning** for your age, but you occasionally have **minor pain** in the joint where the surgery occurred.

1. **Timeline**


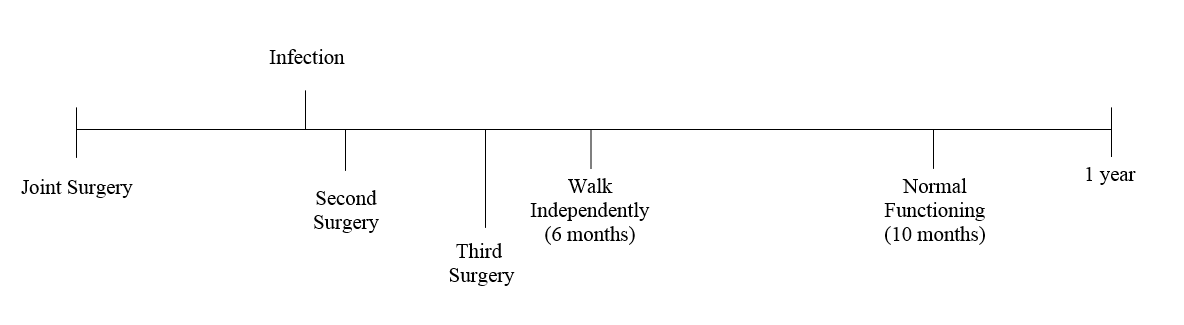


**Health State E. Spine: No Infection**

1. **Surgery**

- You have **spine surgery** to address this problem.
- During surgery, **parts of the bones and soft tissue** surrounding the pinched nerves **are removed** to take the pressure off the nerves.
- **Metalwork** (screws and rods) is inserted to **stabilize** the spine.

1. **Hospital Stay**

- You stay in the hospital for **4 days** after surgery.

1. **After Surgery**

- The surgery itself causes **pain in your back** that **gradually decreases** for about 2 weeks.

1. **Recovery**

- The **original pain** in your legs is **reduced significantly** after the surgery and continues to fade over the next 6 weeks.
- The original pain in your back may improve, but more slowly.
- You use **two crutches** for about 2 weeks. Then, you use **one crutch** for another 2 weeks.
- One month after surgery, you can **walk independently**.
- You continue to improve. **Six months** after surgery, you have **normal functioning** for your age, but you occasionally have **minor pain** in your back where the surgery occurred.

1. **Timeline**


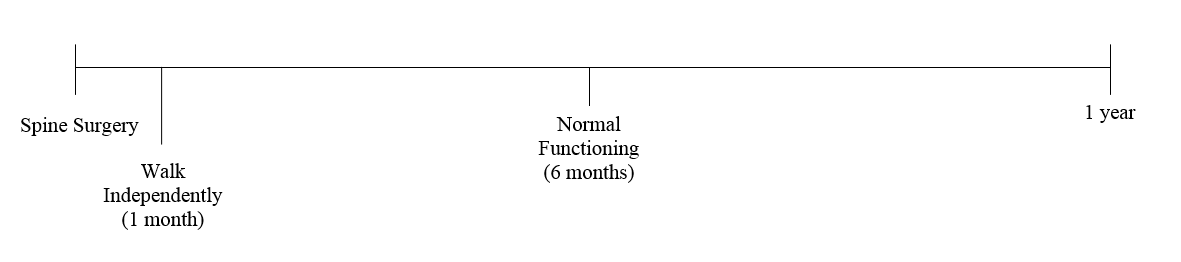


**Health State F. Spine: Infection Not Requiring Surgery**

1. **Surgery**

- You have **spine surgery** to address this problem.
- During surgery, **parts of the bones and soft tissue** surrounding the pinched nerves **are removed** to take the pressure off the nerves.
- **Metalwork** (screws and rods) is inserted to **stabilize** the spine.

1. **Hospital Stay**

- You stay in the hospital for **4 days** after surgery.

1. **After Surgery**

- The surgery itself causes **pain in your back** that **gradually decreases** for about 2 weeks.

1. **Infection**

- Two weeks after surgery, you are diagnosed with a **bacterial infection of the skin**.
- You have **redness and swelling** on the skin surface where the surgery occurred. This area is **hot to the touch**.
- You have some **increased pain and soreness** at the infection site.

1. **Treatment of Infection (Oral Antibiotics)**

- To **treat** this infection, you take **oral antibiotics** multiple times each day for one week.
- For some people, these antibiotics can have many **side effects** including diarrhoea, nausea, and allergic reactions.

1. **Recovery**

- The **original pain** in your legs is **reduced significantly** after the surgery and continues to fade over the next 6 weeks.
- The original pain in your back may improve, but more slowly.
- You use **two crutches** for about 2 weeks. Then, you use **one crutch** for another 2 weeks.
- One month after surgery, you can **walk independently**.
- You continue to improve. **Six months** after surgery, you have **normal functioning** for your age, but you occasionally have **minor pain** in your back where the surgery occurred.

1. **Timeline**


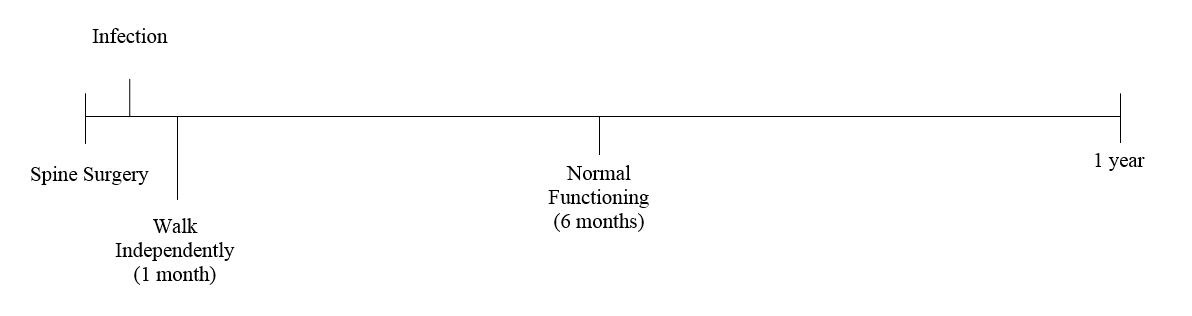


**Health State G. Spine: Infection Requiring Surgery**

1. **Surgery**

- You have **spine surgery** to address this problem.
- During surgery, **parts of the bones and soft tissue** surrounding the pinched nerves **are removed** to take the pressure off the nerves.
- **Metalwork** (screws and rods) is inserted to **stabilize** the spine.

1. **Hospital Stay**

- You stay in the hospital for **4 days** after surgery.

1. **Infection**

- Two weeks after surgery, you are diagnosed with a **deep wound infection**. This involves the **soft tissue** and metalwork.
- You have **redness, swelling, and draining fluid** where the surgery occurred. This area is **hot to the touch**.
- Because of this infection, you have **significantly increased, deep, and persistent pain** in your back.
- The **fluid draining** from the infection **stains** your clothing and bedsheets.

1. **Treatment of Infection (Second Surgery and IV Antibiotics)**

- You need **one additional surgery** to treat the infection.

**SECOND SURGERY**

- During surgery, any **soft tissue** that appears **infected** is **removed**.
- A **temporary tube** is inserted so that bacterial fluid can **drain out** of the wound while you are in the hospital. This tube is removed within the first 2 days after surgery.
- You stay in the hospital for **7 days**.

**MEDICATION AFTER SECOND SURGERY**

- In the hospital, you receive **antibiotic treatment** about 4 times per day by **intravenous (IV) infusion** in the hospital. This means it is administered into the bloodstream through a tiny plastic tube inserted directly into a vein. This takes about 30 minutes.
- After leaving the hospital, you continue to receive antibiotic treatment by IV once per day for 6 weeks. A **district nurse** will come to your home to administer treatments and each time, the treatment process takes approximately 1 hour.

1. **Recovery**

- After the second surgery, the **original pain** in your legs is **reduced significantly** and continues to fade over the next 6 weeks.
- The original pain in your back may improve, but more slowly.
- You use **two crutches** from the time of the first surgery until 2 weeks after the second surgery. Then, you use one **crutch** for about the next month.
- Six weeks after your original surgery, you can **walk independently**.
- You continue to improve. **Eight months** after the original surgery, you have **normal functioning** for your age, but you occasionally have **minor pain** in your back where the surgery occurred.

1. **Timeline**


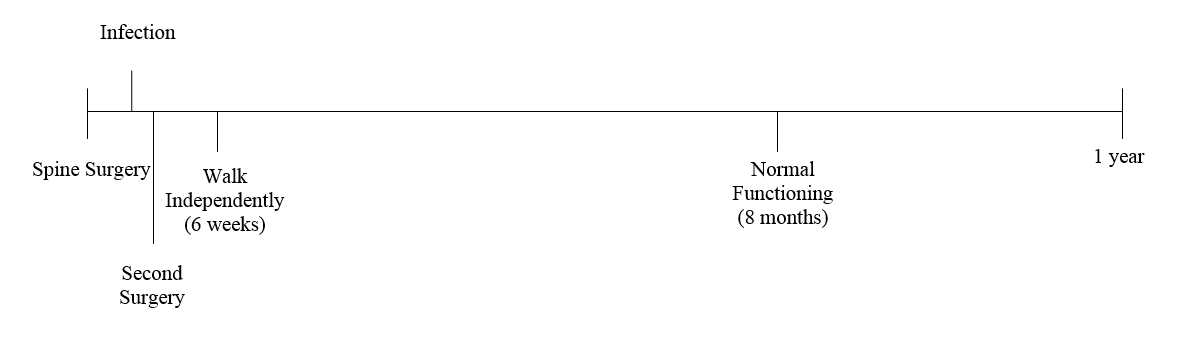

Supplement: Supplementary file 1 — Supplementary material 1 (DOCX 99 KB) [file 10198_2019_1036_MOESM1_ESM.docx]
